# SVBE: searchable and verifiable blockchain-based electronic medical records system

**DOI:** 10.1038/s41598-021-04124-8

**Published:** 2022-01-07

**Authors:** Norah Alrebdi, Abdulatif Alabdulatif, Celestine Iwendi, Zhuotao Lian

**Affiliations:** 1 Department of Information Technology, College of Computer, Qassim University, Buraydah, 51452 Saudi Arabia; 2 Department of Computer Science, College of Computer, Qassim University, Buraydah, 51452 Saudi Arabia; 3 School of Creative Technologies, University of Bolton, Bolton, UK; 4 University of Aizu, Aizuwakamatsu, Japan

**Keywords:** Assay systems, Health care

## Abstract

Central management of electronic medical systems faces a major challenge because it requires trust in a single entity that cannot effectively protect files from unauthorized access or attacks. This challenge makes it difficult to provide some services in central electronic medical systems, such as file search and verification, although they are needed. This gap motivated us to develop a system based on blockchain that has several characteristics: decentralization, security, anonymity, immutability, and tamper-proof. The proposed system provides several services: storage, verification, and search. The system consists of a smart contract that connects to a decentralized user application through which users can transact with the system. In addition, the system uses an interplanetary file system (IPFS) and cloud computing to store patients’ data and files. Experimental results and system security analysis show that the system performs search and verification tasks securely and quickly through the network.

## Introduction

With digital transformation and the appearance of some new concepts in the medical field, such as the Internet of Medical Things (IoMT), it has become important to digitize medical records completely. Electronic medical records (EMRs) provide several advantages, such as ease of accessibility from any device at any time, more efficiency, and saved time and cost. EMRs also enable medical organizations to adopt IoMT technologies, which can help effectively monitor patients statuses remotely and in real time, in addition to faster medical data processing. However, EMR systems face multiple challenges due to several aspects, such as security issues, privacy issues, and technical issues. Hence, EMR systems need a robust design to overcome these challenges and achieve the desired goals.

Many organizations and governments have adopted EMR systems based on cloud computing. Cloud computing provides many features, such as on-demand access, ubiquity, flexibility, and extensibility. The National Institute of Standards and Technology (NIST) defines cloud computing as “a model for enabling ubiquitous, convenient, on-demand network access to a shared pool of configurable computing resources (e.g., networks, servers, storage, applications, and services) that can be rapidly provisioned and released with minimal management effort or service provider interaction”^1^. Despite the several advantages presented by cloud computing, the cloud suffers from central management. Centralizing management of the health records stored in the cloud may result in tampering, forgery, or unauthorized publication.

Several studies have proposed an EMR system based on blockchain techniques. Generally, a blockchain is a directed acyclic graph (DAG); it contains many blocks, and each block is connected with the preceding block by a hash^2^. Blockchain is regarded as a distributed ledger technology (DLT), which refers to storing distributed records and securing them using consensus protocols^3^. Moreover, blockchain is a decentralized technique, which means it does not require reliance on a third party to perform transactions and build trust between users. Additionally, the blockchain transaction record cannot change, thus ensuring untampered data. However, blockchain is characterized by transparency, which can threaten transaction privacy. In addition, storing large data is a major challenge in a blockchain environment^4^.

The different blockchain functions and threats have made it challenging to adopt a specific blockchain methodology for EMRs. Consequently, the features in the proposed blockchain-based systems vary, as some proposals focus on one aspect without the other. Therefore, there is room for new suggestions that provide the multiple functions required for electronic medical systems. This research proposes a new system named the searchable and verifiable blockchain-based EMR System (SVBE). The proposed system will provide the ability to search, verify, and store encrypted EMRs based on blockchain. The main contributions of the paper are as follows:

- Design and implement a new EMR system based on blockchain that provides searchability and verifiability of the encrypted files.
- Apply the interplanetary file system (IPFS) and cloud storage, which help to reduce the costs in the proposed system.
- Conduct a comprehensive investigation of the developed system efficiency through several performance experiments on the following aspects: transaction execution time, medical file sizes, and cost of transactions. A security analysis of the system is also presented.

## Methods

This section introduces the main components. In addition, it illustrates the goals of the system design, the workflow of each function, and the implementation details.

### System architecture

**Figure 1:**
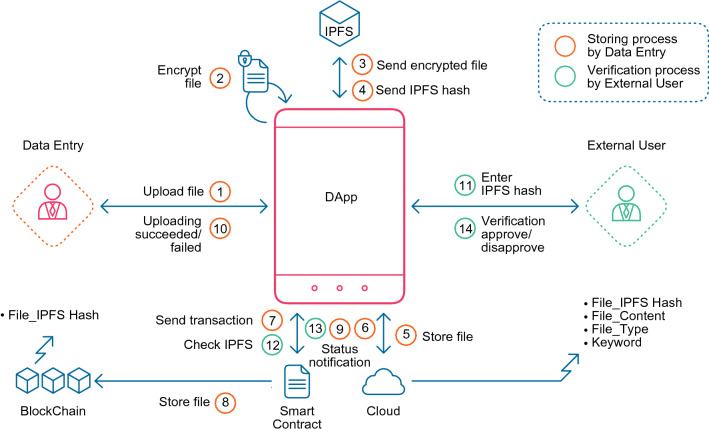
The system architecture of the file storing process conducted by the medical user and the verifying file process conducted by the external user. Several components are used to perform these two functions: SVBE DApp, IPFS, cloud, and blockchain. The stored data are kept in the cloud and blockchain databases.

This subsection presents the SVBE system architecture. Figure 1 illustrates the structure of the proposed system, in which the essential components are shown. The main components are as follows.

#### User application (decentralized application)

This system provides a user application that can be used by medical agents, patients, and external beneficiaries. The application offers two functions related to the patients (add patients and verify patients) in addition to three functions related to the patient records. In this work, three functions related to the patient records are introduced. The first function adds the patient medical files. The second function is a validation service for the stored files that any external beneficiaries can use without worrying about tampering with original data based on the blockchain characteristics. The third function searches for any medical file stored in the system. One of the most important application tasks is to encrypt the uploaded files using the asymmetric cryptography algorithm ECC.

#### Interplanetary file system (IPFS)

Medical systems usually deal with files that can be rather large, which causes difficulty in storing them directly on the blockchain. Hence, the SVBE system needs to deal with a reliable storage environment. The system adopted the IPFS decentralized environment to store the patients medical files. IPFS is considered a suitable solution for dealing with medical files due to several advantages, such as being free from a single point of failure and having high throughput in storage. An IPFS generates a unique hash for each stored file, giving users the ability to find that file by the hash address.

#### Cloud computing storage

The proposed SVBE system relies on storing all data in the cloud. The system stores all encrypted patient data such as personal data, file type, and the keywords of the stored files, in addition to the IPFS hash of the encrypted file. In addition, the system stores the actual content of the medical files after encrypting them to maintain data privacy.

#### Blockchain network

As mentioned earlier, since it is difficult to store large files in a blockchain environment, the system restricts the storage to the IPFS address of the medical files and all the encrypted patient personal information in the blockchain environment. The whole encrypted file is stored in the cloud.

### Design goals

To be effective, the proposed system must achieve the following design goals:

- The system should ensure the security and privacy of the patient records. Therefore, the system has to apply strict rules to guarantee data integrity and confidentiality, and the system needs to prevent access to the records by any unauthorized entities.
- The system should have the ability to search for any file or patient despite the encryption.
- The system should provide the ability to verify the files by any outside related entity.
- The system must achieve high performance and low costs regarding latency, storage, and price to be suitable for adoption in the medical sector.

### Workflow

The system provides three essential functions. The first function is storing a file in the patient record. The second function is verifying from any stored file. Finally, the third function is searching. The three functions are available only for the authorized health agents, except the verification function is available to any external entity. Each functional workflow is described in detail in the following subsections.

#### Files addition function

To add a new file, it is required that the patient who owns the file has a previous record in the system. Blockchain databases are not suitable for storing extensive data. To decrease the blockchain overhead, fewer data are stored in the blockchain database compared to the data stored in the cloud database. Thus, blockchain and cloud require different inputs sent by the Dapp based on the user inputs. The inputs of each party are explained as follows.

The following tuple shows the data that our system’s decentralized application requires for adding a new file:

1 $$\begin{aligned} AddingFile_{DAPP}=(P_{id}, T_{f}, K_{w}, f) \end{aligned}$$

where:

$P_{id}$ = means the patient’s identification number.

$T_{f}$ = the type of file, whether it is a report, medical test, medical history, etc.

$K_{w}$ = keywords that relate to the file content that can use it in the search process.

$f$ = a new file of a patient that will store in a medical records system.

The following tuple describes the data that are sent to the blockchain upon adding a new file:

2 $$\begin{aligned} AddingFile_{BC}=(P_{id},f_{hash}) \end{aligned}$$

where:

$f_{hash}$ = hash of the file generated from IPFS.

The following data are sent to the cloud:

3 $$\begin{aligned} AddingFile_{C}=(P_{id},f_{hash},T_{f}, K_{w},f_{ec}) \end{aligned}$$

where:

$f_{ec}$ = encrypted file content.

**Figure 2:**
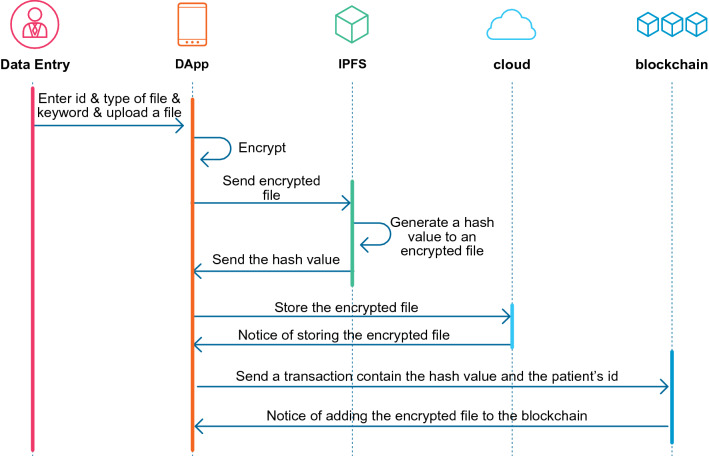
A sequence diagram of the process of adding files process to the system. The process starts by uploading a file, encrypting it, sending it to the IPFS to generate a hash, and then storing it in the cloud and blockchain.

As evident, the system excludes the entire content of the encrypted file stored in the blockchain due to the difficulty of storing large files or data in the blockchain database. Hence, it compensates by storing the encrypted file in the cloud. Storing the encrypted file in the cloud helps the medical user access and retrieve files directly from the cloud by entering the file IPFS hash. Figure 2 shows the workflow of the file adding process.

#### File verification function

The system provides a service to verify files circulating between users to prevent fraud and forgery. An IPFS hash is stored for each file exported from the medical system. Therefore, during the verification process, the user must enter the patient’s ID in addition to uploading the file to be verified. The following tuple shows this step:

4 $$\begin{aligned} VerifyFile_{C}=(P_{id},f_{ec}) \end{aligned}$$

When the file is uploaded, all the steps applied when uploading a new file are performed, which means that if this file is trusted, it will get the same IPFS hash of the original file stored in the system. When the Dapp sends the IPFS hash to the blockchain, the blockchain informs the system that this file is identical to one of the files stored in the patient’s record. Hence, a message will appear to the user stating that this file is trusted. In case of the file is forged or modified, its IPFS hash will not match any of the files stored in the patient record in the blockchain, and a message will appear to the user stating that the file is not trusted. The verification process relies upon the blockchain rather than the cloud because the blockchain environment is more reliable.

#### Search function

Search is a required feature to make the system more functional. The medical user can search for any patients file they need by entering two mandatory inputs: the type of file and the keyword who wants to search for. The time range of the patient birth year is an optional input to narrow the search. If the medical user wants to narrow the search, they need to enter the time range they want to search through. The following tuple explains the search process.

5 $$\begin{aligned} Searching File_{DAPP}=(T_{f}, K_{w}, B_{starty}, B_{endy}) \end{aligned}$$

where:

$B_{starty}$ = demotes the start of the search time range.

$B_{endy}$ = demotes the end of the search time range.

**Figure 3:**
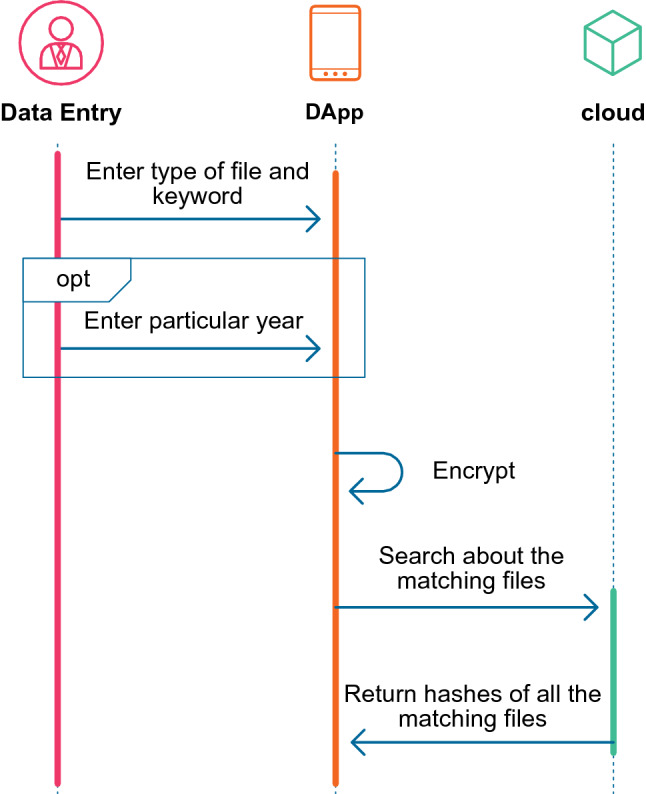
A sequence diagram of a search process in the system through which file type and the keywords are entered (additionally, it is possible to enter the time range), encrypted, and searched in the cloud, which together generate the results.

Figure 3 shows the search function. The search is performed directly by searching in the cloud database to reduce the costs involved in the blockchain environment. Moreover, an inverted index technique is used to index the keywords in the cloud to reduce the search cost. An inverted index form is a pair (key, value), where the key is a keyword and the value is a list of IPFS hash of files related to the keyword. Table. 1 shows the architecture of the files linked with the keywords in the cloud. The search results can be customized either by returning the file hash or the patient’s ID.

**Table 1:** Structure of the cloud-based proposed records system.

Key	Value
Rachitis	IPFS Hash of file 1, file 2,.
Allergies	IPFS Hash of file 3,.
Cancer	IPFS Hash of file 4, file 2,.

### Implementation

The detailed implementation and tools used in the proposed system are presented. Additionally, the system design and description of the data stored in the cloud and blockchain databases are illustrated in the following subsections.

#### System settings

The proposed system consists of several parts: the user application, smart contract blockchain, IPFS, and the cloud database. For the blockchain, a simulated public Ethereum network is used. The model uses a local Ganache to run a local blockchain. Ganache provides 10 accounts, each with 100 ethers that are used to conduct the transactions. These accounts can be used by using MetaMask, a web browser extension that links the application with the smart contract. The smart contract was written in the Solidity language using the Remix code editor. The user application was written in Javascript. The system uses a React library to build the user frontend. The node package manager (NPM) of ECC was used to encrypt the data. To link the application with the blockchain network, the WebJs3 library was used, which is a library that interacts with MetaMask in the application. Furthermore, the system uses IPFS to generate a hash for each medical file. For cloud storage, the system uses two Firebase products: Cloud Firestore and Cloud Storage, to store both separate data and medical record files.

#### System design

A smart contract was built for the system, which the system administrator monitors. The smart contract conducts several functions, including add_file and verify_file. The pseudocode of these two functions is explained in Algorithm 1. The search function is conducted on the cloud database. The pseudocode of the search function is described in Algorithm 2.

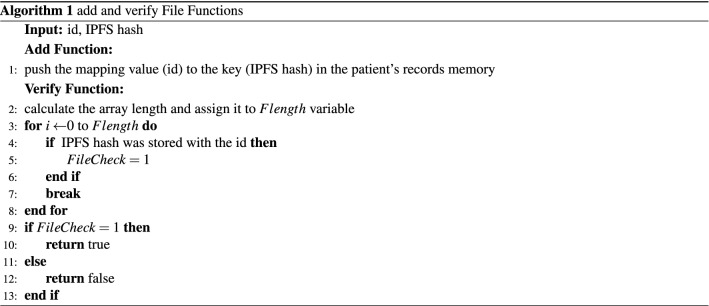

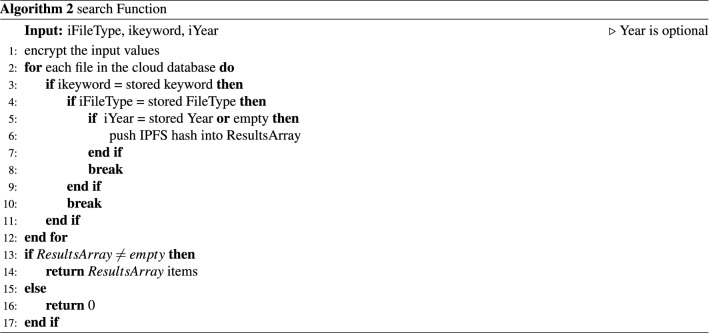





#### Cloud-based data storage

The proposed system uses two Firebase products: Cloud Firestore and Cloud Storage. Cloud Firestore is a NoSQL database that accepts several data types. The Cloud Firestore database is organized as a hierarchical structure. The root of Cloud Firestore is a collection; each collection has many documents depending on the stored data. Then, each document has several attributes. There are three collections in Cloud Firestore: patients, files, and keywords. Storing any patient on the patient collection requires five fields: ID, first and last name, and birthdate. The system stores the birth year as a separate attribute to allow an encrypted search for patients of a specified age. File collection contains two fields: patient ID, which links the file with its owner, and the IPFS hash of the file. The keyword collection includes two values: the keywords and the list of the IPFS hashes. The keyword is stored in a document, and each keyword document contains a list of IPFS hashes of files associated with the keyword. Cloud storage is a storage service suitable for storing files, voices, or images. Cloud storage stores the files and provides information about them, namely, the filename, size, type, and last modification. Cloud storage is used to store patient medical documents and images. Each file name with its IPFS hash to link each file with its owner’s medical record.

#### Blockchain-based data storage

There are fewer data stored in the blockchain than data stored in the cloud. Generally, the data stored in the blockchain are divided into two parts: one for the patients and the second for the files. The patient part stores four attributes, which are the same as those stored in the cloud: ID, first and second name, and birthdate. Then, the file part stores two attributes: a patient’s ID connected with the list of all IPFS hashes of its files.

## Results

In this section, the proposed system is discussed in two aspects. The first aspect is system performance, where several experiments were conducted to calculate the transactions’ latency, stored file sizes, and transactions’ costs. For the credibility of the results, the average of 10 experiments of each function is shown. Each of the three aspects is discussed individually in a subsection. For the second aspect, the security of the system is analyzed.

### Transactions latency

**Figure 4:**
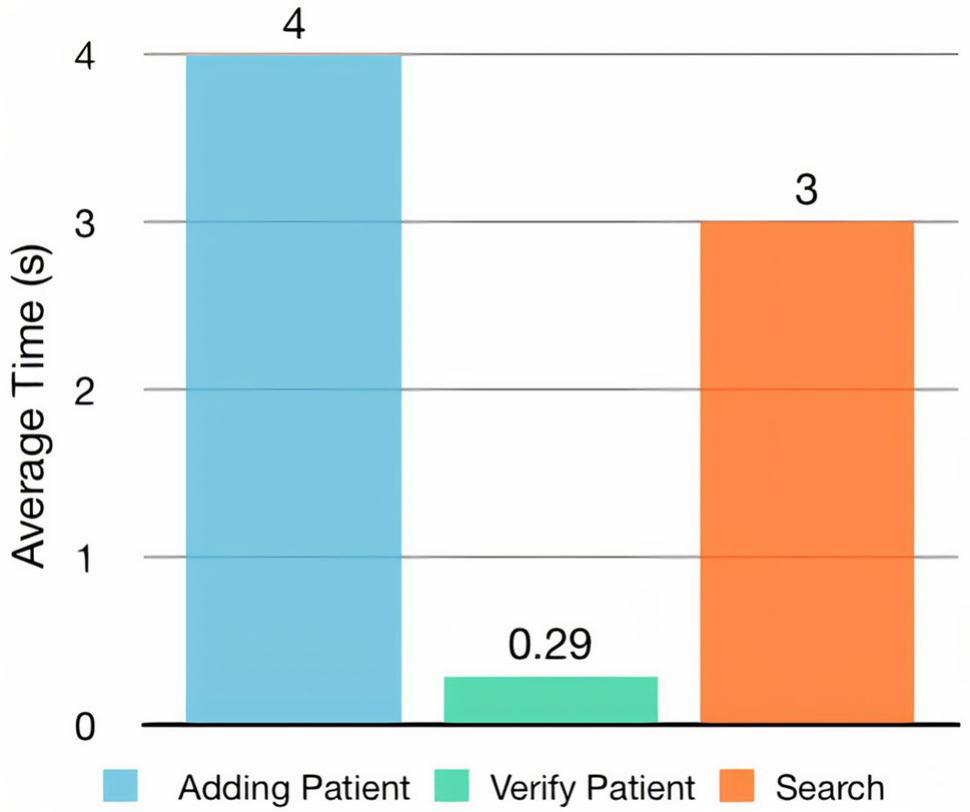
Transaction latency of functions.

The time taken to complete the functions provided is an essential factor in evaluating system performance. Therefore, several latency calculations were performed that showed the performance of the proposed system. Figure 4 shows the average processing time of the patient adding function, patient verifying function, and search function. The table data shows that the verification time was the shortest, where it took fractions of a second. Furthermore, the time taken in the search function was less than that of adding patients. All three functions took a short amount of time. Notably, adding file function is conducted by using both databases: blockchain and cloud databases. In contrast, the verifying file function is conducted using the blockchain database only, while the search function is conducted using the cloud database only. Moreover, the patient adding function requires an initial step to ensure that the patient does not already exist in the system; this may explain the long time it took by the add patient function compared with the verification function.

**Figure 5:**
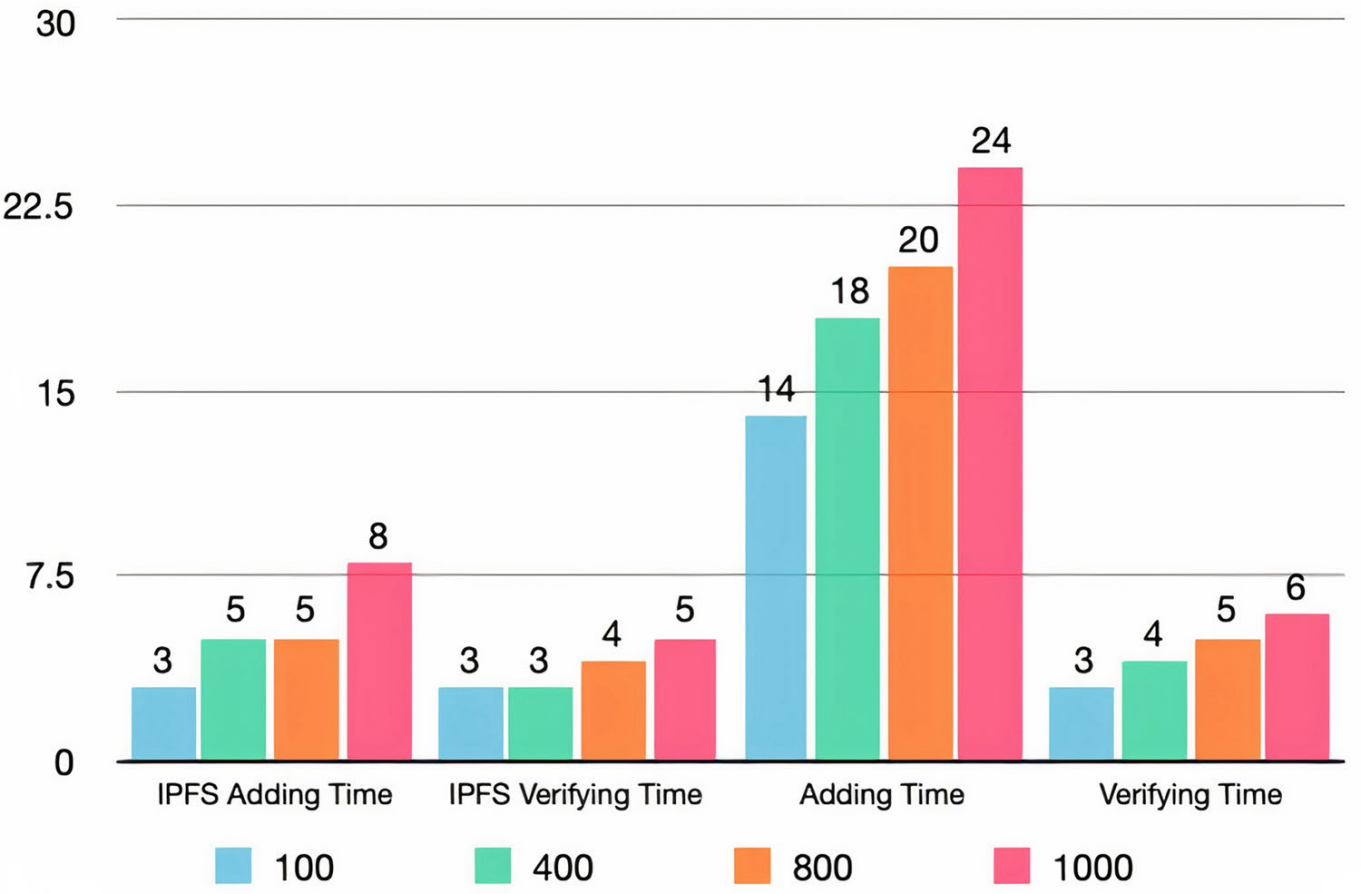
Transaction latency of adding and verifying files.

Moreover, adding and verifying file functions were tested on four different file sizes. In addition, the time taken for the adding and verifying file processes on IPFS was calculated separately. Figure 5 shows the latency results of adding and verifying files in both situations. The figure shows that the latency of adding and verifying files usually increased with the increase in file size. However, the verification process through the IPFS took the most time during the four experiments, such that the entire verification process required 0–1 s more than the IPFS verification. The longest time was 24 s, which was associated with the file adding process taken with a file size of 1000 kilobytes (kB). Therefore, the transaction latency calculations were logical. Thus, this test achieved the fourth goal of the system design goals.

### File size

**Figure 6:**
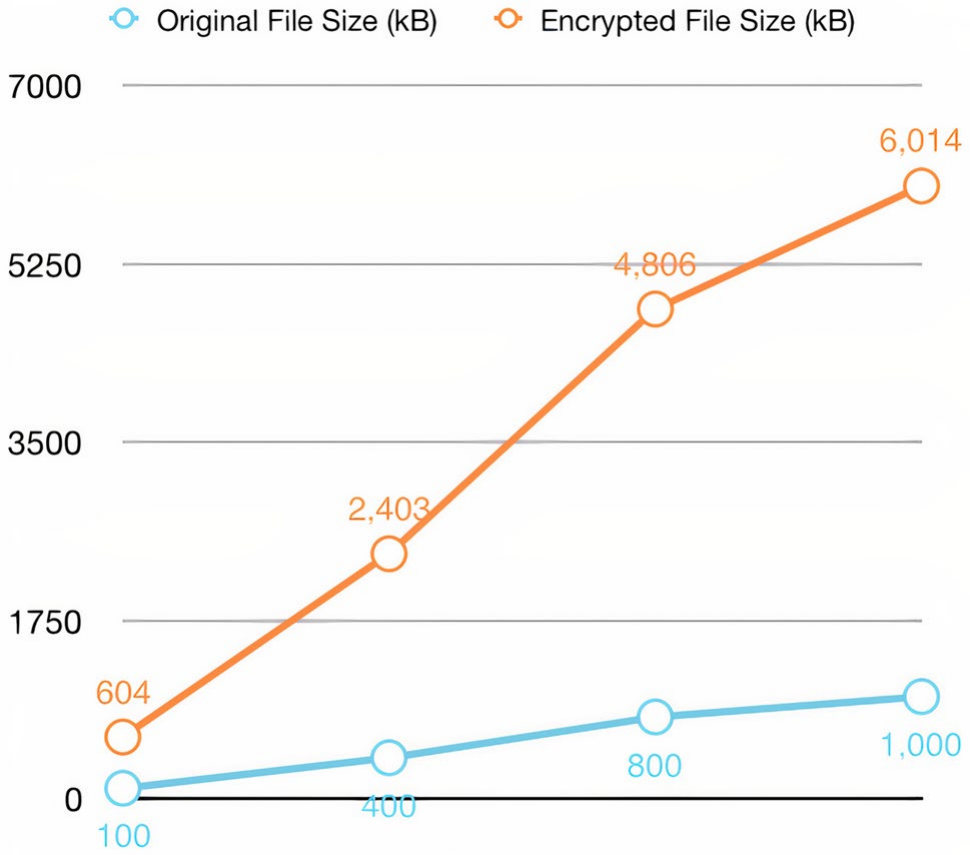
Comparison of the file size before and after encryption. The blue line illustrates the unencrypted file size, and the orange line indicates the encrypted file size.

The size of medical files varies and may tend to be large. In addition, encryption contributes to increasing the file size, making storing these files in the blockchain a costly process. To solve this problem, only the IPFS hash of the encrypted files is stored in the blockchain. In contrast, the entire encrypted content is stored in the cloud, as the cloud requires lower costs for storing large files. Figure 6 shows the size of the original files and the size after encryption using the elliptic curve cryptography (ECC) algorithm. It is clear from the figure that the file size increases after encryption by six times the original size.

### Transactions costs

In the SVBE system, the functions are divided into two types: call functions and transactions. The transaction is a function that writes on the blockchain or cloud database, while the call function is the function that reads from the databases. Each transaction requires a cost specified based on the number of parameters, the structure of the function in a smart contract, and the used data types. The call function does not have a cost. Both add_patient and add_file functions in the proposed system are considered transactions, and the verify_patient, verify_file, and search functions are call functions. Table 2 shows the average amount of gas used by the two transactions: add_patient and add_file and the costs in US dollars. The value of the conversion used in the table is $2205.65, which is the current value of one ether. For the gas price, the standard value was used which is 20 GWEI. The high cost of the add_patient function may be due to the structure of the function. The add_patient function requires another function responsible for checking if the patient exists in the system beforehand or not. By contrast, the cost of the add_file function is lower than add_patient, which may demonstrate the benefit of storing an IPFS hash of the file instead of the entire file content.

**Table 2:** Transaction cost of adding patients and Files.

Function name	Gas used	Cost (dollar)
Add patient	421,901	18.6
Add file	93,968	4.7

Regarding the experimental results of the system performance, the system provides high performance in the latency aspect. In contrast, the increase in the size of the encrypted files is relatively large, but cloud storage can solve the problem, as it is suitable for storing such sizes. Despite the high cost of adding patient transactions, the system provides several functions that do not require costs. Generally, the experimental results achieve the fourth system design goal. However, the system may need to reduce the cost further to improve the performance in this aspect.

### Security analysis

The proposed system enables the search and verification of medical records securely and privately. The system is secured in several ways, such as using blockchain technology characterized by immutability. The immutability feature prevents any entity from tampering with or modifying the recorded data in the blockchain environment^5^. Thus, the system guarantees the integrity of the stored data^6^. Furthermore, the data entry process is limited to specific entities that the system administrator has to identify previously. Thus, this helps ensure the validity of the entered data and the inability of unauthorized parties to enter any data. In addition, limiting data entry to a certain number of entities makes it possible to investigate the cause when any error occurs in the entry data process. Therefore, these restrictions help to achieve the first system design goal.

Moreover, to ensure the patient privacy, any data and documents must not be stored in plaintext. Thus, the patient’s files and personal data need to be encrypted before storing them in the system. Moreover, the system provides data confidentiality by preventing any unauthorized access or obtaining data explicitly. Thus, only the authorized entities can access the keys that decrypt the patient records and content. Hence, the system does not need to frequently share the keys through the network, protecting the keys from some attacks. Furthermore, the system uses asymmetric cryptography, which protects against eavesdropping attacks. More specifically, the encryption algorithm used in this system is ECC, which provides strong security, although it uses a short key size compared to other encryption algorithms^7^. Moreover, the encrypted files are stored in decentralized IPFS. The decentralization characterized by blockchain and IPFS protects against a single point of failure. In addition, decentralization is a peer-to-peer method, meaning it does not need trust in any third party to conduct transactions.

Regarding the search function, providing a search function using multiple keywords may lead to disclosing some data or guessing it, threatening patient data privacy. The system provides the search function using only one keyword to preserve privacy, although it can use the time range to obtain more effective results. Additionally, the search results can be controlled and made to contain only the IPFS hashes of the patients files instead of the IDs or the patient names to maintain patient anonymity. Thus, this achieves the second system design goal.

The system provides the file verification function to enable the permitted external entities to ensure the authenticity of the medical files even though they are encrypted. Consequently, the user who wants to verify a file does not need to obtain the plaintext file, as the ciphertext can do the job. Thus, this maintains patient data privacy and achieves the third goal of the system design goals, which is reduced patient privacy exposure.

Performance and security analysis show that the proposed system achieves the required design goals. In addition, the system has many features, such as flexibility and availability, so that different devices and systems can use it.

## Discussion

Multiple studies have proposed several solutions to address the attacks and security threats based on different technologies^8,9^. For example, Tan et al.^10^ introduced an approach called HoneyNet that includes threat detection and situational awareness of the artificial intelligence of things. Furthermore, information-centric networking was used to enhance communication security in smart grid^11^. Tan et al.^12^ developed a traceable and direct revocation schema for medical records. In contrast, to enhance the communication performance, three types of nonlinear RF chain structures that reduce the power consumption of multiple-input multiple-output wireless communication systems were designed in^13^.

Furthermore, several techniques are used to preserve data privacy. For example:^14^ used attribute-based encryption (ABE) integrated with the 0-1 coding technology to enhance the encryption performance of the internet of health things data. Hang et al.^15^ used ABE with a parallel outsourced decryption method. Additionally, a new method of secure arrangement based on matrix eigenvalue calculation was proposed in^16^. Moreover, the authors in^17^ proposed enhanced retrieval models between the IoT and the cloud.

Regarding the blockchain-based systems, Wang et al.^18^ proposed a new certificateless signature scheme integrated with the blockchain. Additionally, Xiong et al.^19^ developed an efficient blockchain batch verification scheme using the elliptic curve digital signature algorithm. Moreover, Liu et al.^20^ proposed a secure framework that used blockchain with mobile-edge computing to provide secure data sharing. Additionally, several studies have proposed medical records systems based on a blockchain. For instance, Liu et al.^21^ designed a system that improves diagnosis processes in electronic health systems. Rahman et al.^22^ introduced a tamper-proof health electronic record management system. Instead of using a blockchain to store health records, a blockchain-based healthcare system has been proposed to store addresses of mobile devices and sensors for the pervasive social network (PSN)^23^. Similarly, Xia et al.^24^ stored the URLs of Fast Healthcare Interoperability Resources (FHIR) instead of the actual medical records. In contrast, a cloud-based electronic health records system was proposed based on blockchain and the IPFS^25^. Encrypted files are stored in the IPFS and linked to the blockchain through a patient ID and address. In addition, Jabarulla and Lee^26^ proposed a blockchain-based management system to store and share medical images securely. The authors in^27^ proposed a system for sharing medical data that guaranteed patient privacy by allowing only authorized parties to use it. Moreover, a new system was proposed in^28^ to securely share records in emergency states using a multiparty computation circuit. In contrast, to enhance patients’ control of their data, Xia et al.^29^ suggested a system that allows the patients to give or restrict the access permissions of their files.

Searchable symmetric encryption (SSE) was first introduced by Song et al.^30^ to secure searching on encrypted data. Curtmola et al.^31^ used a single keyword in search. Chen et al.^32^ developed a search method using multiple keywords. Moreover, Cash et al.^33^ proposed an SSE framework that concentrates on conjunctive search and Boolean queries. As an extension of Cash et al.’s work^33^, Faber et al.^34^ developed more capabilities: search in a range, wildcard, substring, and phrase queries. The authors in^35^ proposed a solution for keyword typos based on the fuzzy search algorithm. Kamara et al.^36^ provided the SSE schema using an inverted index. The red-black tree index was used in^37^. A tree-based index schema was introduced by Xia et al.^38^ and conducted on encrypted cloud data. Moreover, Xiru et al.^39^ proposed a searchable system of the encryption contents using keywords binary tree scheme to enhance the searchability in the blockchain. Similarly, some studies used SSE with blockchain to enhance search efficiency and ensure stored data privacy, such as in^40,41^. Several EMR systems based on a blockchain environment have been introduced. Moreover, some studies have provided search features in their blockchain EMR system. However the published studies do not propose a medical system that provides both features: the ability to search for encrypted medical files and the ability to verify them. This paper presents a design of an electronic medical records system that ensures privacy, security, searchability, and verifiability based on the blockchain technique.
